# SARS-CoV-2 Associated Pediatric Inflammatory Multisystem Syndrome With a High Prevalence of Myocarditis – A Multicenter Evaluation of Clinical and Laboratory Characteristics, Treatment and Outcome

**DOI:** 10.3389/fped.2022.896252

**Published:** 2022-06-10

**Authors:** Katharina Thom, Beatrice Kahl, Thomas Wagner, Andreas van Egmond-Fröhlich, Mathias Krainz, Thomas Frischer, Iris Leeb, Christine Schuster, Doris Ehringer-Schetitska, Milen Minkov, Christoph Male, Ina Michel-Behnke

**Affiliations:** ^1^Department of Pediatrics, Division of Pediatric Cardiology, Pediatric Heart Centre, Medical University of Vienna, Vienna, Austria; ^2^Department of Pediatrics and Adolescent Medicine, Clinic Donaustadt, Vienna, Austria; ^3^Department of Pediatrics and Adolescent Medicine, Clinic Favoriten, Vienna, Austria; ^4^St. Anna Children’s Hospital, Center for Children and Adolescent Medicine, Medical University Vienna, Vienna, Austria; ^5^Department of Pediatrics and Adolescent Medicine, Clinic Ottakring, Sigmund Freud Private University, Vienna, Austria; ^6^Department of Pediatrics and Adolescent Medicine, State Hospital Mödling, Mödling, Austria; ^7^Department of Pediatrics and Adolescent Medicine, State Hospital Mistelbach-Gänserndorf, Mistelbach, Austria; ^8^Department of Pediatrics and Adolescent Medicine, State Hospital Wiener Neustadt, Wiener Neustadt, Austria; ^9^Department of Pediatrics and Adolescent Medicine, Clinic Floridsdorf, Vienna, Austria

**Keywords:** COVID-19 associated pediatric inflammatory syndrome, myocarditis, cardiac decompensation, inotropic support, COVID-19, PIMS, echocardiograghy

## Abstract

**Introduction:**

*Pediatric inflammatory multisystem syndrome – temporally associated with SARS-CoV-2 infection (PIMS –TS)* comprises a new disease entity having emerged after the COVID-19 outbreak in 2019.

**Materials and Methods:**

For this multicenter, retrospective study children between 0 and 18 years with PIMS-TS between March 2020 and May 2021 were included, before availability of vaccination for children. Frequent SARS-CoV-2 variants at that period were the wildtype virus, alpha, beta and delta variants. Inclusion criteria were according to the PIMS-TS criteria, proposed by the Royal College of Pediatrics and WHO. Study aim was to review their clinical, laboratory and echocardiographic data with a focus on cardiac involvement.

**Results:**

We report 45 patients, median age 9 years, 64% male. SARS-CoV-2 antibodies were positive in 35/41 (85%). PIMS occurrence followed local COVID-19 peak incidence periods with a time lag. The most common symptoms at presentation were fever (98%), abdominal pain (89%) and rash (80%). Fever history of > 5 days was associated with decreased left ventricular function (*p* = 0.056). Arterial hypotension and cardiac dysfunction were documented in 72% patients, increased brain natriuretic peptide in 96% and increased cardiac troponin in 64% of the children. Echocardiography revealed mitral valve regurgitation (64%), coronary abnormalities (36%) and pericardial effusions (40%). Increased NT-proBNP was significantly associated with the need of inotropics (*p* < 0.05), which were necessary in 40% of the patients. Treatment comprised intravenous immunoglobulin (93%), systemic steroids (84%) and acetylsalicylic acid (100%; 26/45 started with high dosages). For insufficient response to this treatment, five (11%) children received the interleukin-1 receptor antagonist anakinra. All patients were discharged with almost resolved cardiac signs.

**Conclusion:**

Our analysis of non-vaccinated children with PIMS-TS demonstrates that a considerable number have associated myocarditis requiring intensive care and inotropic support. Most children showed adequate response to intravenous immunoglobulin and steroids and good recovery. Further evaluation of pediatric patients with COVID-19 associated diseases is required to evaluate the impact of new virus variants.

## Introduction

*Pediatric inflammatory multisystem syndrome – temporally associated with SARS-CoV-2 infection (PIMS –TS)* in Europe and *Multisystem Inflammatory Syndrome in Children (MIS-C)* in the United States comprises a new disease entity that emerged after the COVID-19 outbreak in 2019. Available literature from several countries reveals similarities to Kawasaki disease (KD), viral myocarditis and toxic shock syndrome, but also differences. Patients with PIMS-TS tend to be older than KD patients with a median age of 9 years. The majority of prior reported children had a COVID-19 infection 2–6 weeks before PIMS-TS and commonly presented with high fever, rash, and abdominal symptoms (1–6).

More than 70% of patients develop myocarditis with dyskinetic, impaired ventricular function, valve regurgitation, rhythm disturbance, and pericardial effusion. The coronary arteries may show perivascular echogenicity progressing to coronary aneurysms in up to 14% of the cases. Patients with PIMS-TS associated myocarditis are at increased risk for severe hypotension or cardiogenic shock, and may require intensive care treatment, inotropic support and rarely invasive cardiac support (7–10).

In the autumn of 2020 several Austrian pediatric departments noted an increasing occurrence of cases with a COVID-19 infection-associated inflammatory syndrome and myocarditis. The aim of this study was to evaluate PIMS-TS cases in Austria to describe clinical and laboratory characteristics at presentation with special focus on cardiovascular involvement, therapy and outcome.

## Materials and Methods

### Study Population

The investigation included 45 consecutiv pediatric patients, age 0–18 years, diagnosed with PIMS-TS between March 2020 and May 2021 according to PIMS-TS diagnostic criteria by Royal College of Pediatrics and Child Health and WHO (11, 12). The cohort comprised all patients with PIMS-TS admitted to the Department of Pediatrics, Medical University of Vienna and eight secondary care pediatric departments in Vienna and Lower Austria. All departments had local ethics board approval for this data collection, data were deidentified, centrally connected to patient identification numbers.

### Data Assessment

Clinical and diagnostic data were obtained from electronic medical records and entered in a case report form specifically designed for this study to ensure systematic data collection.

*Data assessment included:*

(i) Chart review regarding medical history and clinical status and medical treatment. “History of COVID-19 infection” was defined – according to WHO and RCPCH criteria as positive SARS-CoV-2 antigen or polymerase chain reaction (PCR) test or exposure to a positive tested person within the prior weeks (11, 12). “Asymptomatic COVID-19 infection prior to PIMS-TS diagnosis” applied to children who had SARS-CoV-2 antibodies at presentation and reported contact to a COVID-19 positive tested person within the prior weeks, but never had had symptoms.
(ii) Laboratory results: complete blood counts and chemistry including C-reactive protein (CRP), Interleukin-6 (IL-6), ferritin (documentation of normal or abnormal), and coagulation parameters. Cardio-specific biomarkers: N-terminal pro-B type natriuretic peptide levels (NT-proBNP, measured by ChemiLumineszenz ImmunoAssay, Fa Roche, pg/mL), cardiac troponin T or I (TnT, TnI, high-sensitivity method; normal < 14 ng/L), documented as measured at the respective departments. Cardiac troponin was documented as normal or abnormal (no levels).
(iii) Microbiology: On admission all patients had a nasopharyngeal swab for viral polymerase chain reaction of SARS-CoV-2 done (PCR Genexpert Roche COBA 6800) and testing for SARS-CoV-2 nucleocapsid IgG antibodies to viral spike glycoprotein in this not vaccinated cohort (e.g., qualitative Elecsys^®^ Anti-SARS-CoV-2 Test, Roche). Additionally, viral myocarditis work-up and bacterial cultures were performed. No COVID-19 antigen (rapid) tests were included.
(iv) Review of cardiac diagnostics included electrocardiography (ECG), echocardiography and cardiac magnetic resonance imaging (MRI). Further imaging comprised abdomen or kidney ultrasound, if performed based on clinical presentation.

Response to treatment was defined as decline of fever and abdominal pain, normalization of CRP and declining NT-pro BNP within 3–5 days of therapy initiation.

### Statistics

Data on patient characteristics, clinical features, diagnostic results, therapy and outcome were summarized descriptively. Continuous variables were expressed as median, interquartile ranges (IQR), or minimum and maximum (min; max); categorical variables as numbers and percentages. For analysis of associations we used the Chi Quadrat Test and Students t-test, respectively.

## Results

### Patient Characteristics

**FIGURE 1:**
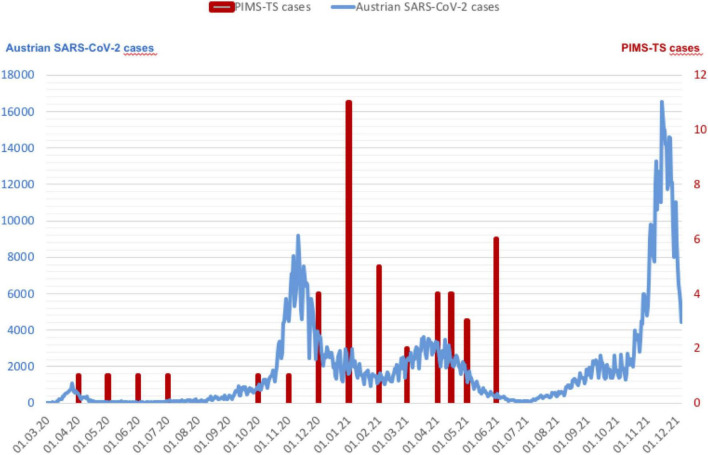
The Graph demonstrates patients with PIMS-TS and Austrian COVID-19 cases (*blue curve*, data from Statistic Austria) (29). Of the 45 children 39/45 (87%) patients presented with PIMS-TS between October 2020 and January 2021 (*n* = 20) and February to May 2021 (*n* = 19). The remaining 6/45 (13%) cases were reported between April and September 2020 (*red bars*).

Forty-five patients with PIMS-TS were treated in the participating centers from March 2020 to May 2021 and included in the study. Their median age was 9 years, (IQR 4–12), 64% male. Further patient characteristics are given in Table 1. A history of COVID-19 infection was reported in 37/45 (82%). Thirty-nine/45 (87%) patients presented with PIMS-TS between October 2020 and January 2021 (*n* = 20) and February to May 2021 (*n* = 19), following local Austrian SARS-CoV-2 infection peaks, with a time lag of 3–6 weeks. The remaining 6/45 (13%) cases were reported between April and September 2020 (Figure 1).

**TABLE 1:** Demographic data.

Characteristics	*N* (%)	Median (min; max)	Interquartile range (P25–P75)
*Age, years*		9 (1.2;18)	[4–12]
0–5	12 (27)		
6–10	13 (29)		
11–18	20 (44)		
*Sex*			
Male	29 (64)		
Female	16 (36)		
Weight (kg)		31.3 (11;79)	[19–50]
Body mass index (kg/m^2^)		18	[16–20]
*Comorbidity*	6 (13%)		
Recurrent Bronchitis and prematurity	1		
VSD	1		
Hydronephrosis	1		
Latent tuberculosis	1		
Primary immune deficiency (cellular/humoral)	1		
Asthma, atopic dermatitis	1		
*Presentation period*			
April 2020 to September 2020	6 (13)		
October 2020 to January 2021	20 (44)		
February 2021 to May 2021	19 (43)		
Covid-19 positive history (weeks prior PIMS diagnosis)*	37 (82)		
0–2	1 (2)		
2–4	13 (29)		
4–6	13 (29)		
>6**	10 (22)		

*According to positivity of prior antigene or PCR tests or contact-history.

**Maximum 8 weeks. VSD, ventricular septum defect.

#### Clinical Presentation

Table 2 shows leading symptoms at admission. All patients presented in an impaired general condition, fever was present in 44/45 (98%) children, abdominal pain in 89%, and a polymorphic rash in 80% of the patients. Fever history of > 5 days was associated with decreased left ventricular function [11/15 (73%; *p* = 0.056)]. Further symptoms were cough (6%), pharyngitis (15%), and headache (4%). No serious primary respiratory symptoms were reported.

**TABLE 2:** Signs and symptoms.

Signs and symptoms at presentation	*N* (%)
Impaired general condition	45 (100)
Fever	44 (98)
Gastrointestinal symptoms*	40 (89)
Rash	36 (80)
Pharyngitis, cough	9 (20)
Cerebral/head ache	2 (6)
Arterial hypotension	31 (72)

*Inclusive vomiting, diarrhea, and abdominal pain.

#### Cardiac Abnormalities

Table 3 illustrates the observed cardiac abnormalities. Cardiac dysfunction with severe arterial hypotension and shock was present in 31/45 (72%) children and required admission to an intensive care unit for 14/45 (31%) patients, seven of these required oxygenation, three of those patients were in cardiogenic shock and four children required oxygen because of pleural effusions and pulmonary edema. Chest x-ray revealed signs for pulmonary congestion (11%) and inflammatory changes (24%), but no patient required mechanical ventilation.

**TABLE 3:** Cardiac diagnostic.

Echocardiography and ECG	*N* (%)
Echocardiography generally abnormal	40 (89)
LV dysfunction, decreased LV ejection fraction	15 (33)
Mitral valve regurgitation	29 (64)
Coronary artery abnormalities	16 (36)
Additional pleural effusions	8 (18)
Isolated pleural effusions*	5 (11)
*Electrocardiography*	
Repolarisation abnormalities	12 (27)

LV, left ventricular. *2 with combined pericardial and pleural effusions.

Cardiac work up revealed abnormal echocardiography in 40/45 (89%) patients, including left ventricular dysfunction (33%), mitral valve regurgitation (64%), pericardial effusions (15%), and echogenic walls of the coronary arteries (36%), no coronary aneurysms were detected. Additional pleural effusions were documented in 8/45 (18%) children. Another 5/45 (11%) patients with normal echocardiography results had pericardial effusions (in two patients a combination of pericardial-and pleural effusions) at presentation.

Seven patients had a cardiac MRI performed in the acute phase of PIMS-TS, two revealed pathological results (one had late enhancement of left ventricle posterior wall and one myocardial edema, no fibrosis).

ECG abnormalities were found in 12/45 (27%) children, 8% had ST elevations and 22% T inversion on left precordial leads lasting for 3–8 days. No relevant arrhythmias were detected.

#### Laboratory Results

**FIGURE 2:**
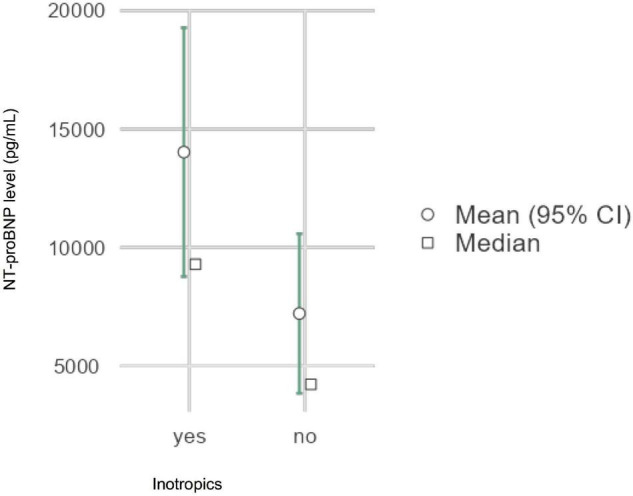
Elevated NT-proBNP levels were associated with the need of inotropics, *p* < 0.05 Inotropics: milrinone (*n* = 6), noradrenalin (*n* = 11), dobutamine (*n* = 1). NT-proBNP: N-terminal-pro-B type natriuretic peptide.

Table 4 summarizes the results of the laboratory work up. The majority of patients (43/45; 96%) showed increased levels of NT-proBNP (median 8876 pg/ml; IQR 2677–10,556) and increased cardiac troponin in 29/45 (64%) patients. Most children (80%) had both cardiac biomarkers abnormal. Increased NT-proBNP was significantly associated with the need of inotropics (*p* < 0.05) (Figure 2). No other associations were found for abnormal NT-proBNP levels or troponin elevation. Markedly increased CRP was present in 37/45 (82%, IQR 14–27), increased Interleukin-6 in 16/35 (46%, IQR 31–1546), and abnormal ferritin in 31/42 (74%) children. Transient impairment of renal function with creatinine elevation was reported in 29% patients in the course of PIMS-TS.

**TABLE 4:** Laboratory results.

Laboratory results	Reference values	Abnormal at admission, *n* (%)	Median	Interquartile range (P25–P75)
*Cardiac-biomarkers*				
NT-proBNP, pg/ml	<125	43 (96)	6477	[2677–10556]
Cardiac troponins ng/L	<14	29 (64)		
*Blood chemistry*				
CRP, mg/dL	<0.5	37 (82)	19	[14–27]
Interleukin-6, pg/mL	<7	16 (46)	181	[31–1546]
Ferritin, mcg/L	6–60	31 (74)		
Creatinin, mg/dL	0.31–0.47	13 (29)		
Fibrinogen (Clauss), mg/dL	200–400	31 (69)	470	[280–550]
*Covid-19 diagnostics at presentation*				
*SARS-CoV-2 PCR*				
Positive		9/44 (20)		
Negative		35/44 (79)		
Not available		1		
*SARS-CoV-2 antibodies*				
Positive		35/41 (85)		
Negative		6/41 (15)		
Not available		4		

*Für NT-proBNP, brain natriuretic peptide; CRP, C-reactive protein.*

*SARS-CoV-2 testing* at presentation revealed positive PCR from nasopharyngeal swab in 9/44 (20%) and COVID-19 IgG antibodies in 35/41 (85%) patients. A “positive COVID-19 history” was documented in 37/45 (82%) children, who all had confirmed COVID-19 IgG antibodies on admission (Tables 1 and 4). Out of 45 patients with PIMS-TS, 17 (38%) had “prior asymptomatic COVID-19 infection” with reported contact to a COVID-19 positive tested person within 2–6 weeks prior to admission, no symptoms or tests in the past, but positive SARS-CoV-2 antibodies at presentation/on admission.

#### Treatment

Table 5 summarizes the treatment of our cases. Severe myocardial dysfunction and arterial hypotension required therapy with inotropic agents in 18/45 (40%; milrinone: *n* = 6, dobutamine: *n* = 1, norepinephrine: *n* = 11).

**TABLE 5:** PIMS treatment and outcome.

Treatment	*N* (%)
IVIG 1–2 grams/kg over 12–24 h	42 (93)
Corticosteroids	38 (84)
Methylprednisolon (10–30 mg/kg over 3 days)*	24 (53)
Prednisolon (2 mg/kg)	16 (35)
Acetylsalicylic acid	45 (100)
Primary high dose (30–50 mg/kg for 3–5 days)*	26/45 (58)
Low dose (3–5 mg/kg for 3 months)	39/45 (87)
Anakinra (2–4 mg/kg/day)	5 (11)
High fever and gastrointestinal symptoms, exanthema, konjuntivitis	5/5
Arterial hypotension, ICU	3/5; 2/5
Markedly elevated NT-pro BNP; CRP	5/5; 4/5
Prior IVIG	5/5
Prior prednisolone/methylprednisolone	2/5; 3/5
*Inotropes*	18 (40)
Milrinon	6 (13)
Dobutamine	1 (2)
Norepinephrine	11 (24)
*Respiratory support*	
Oxygen intranasal insufflation	7 (16)
*Treatment response* (improved clinical, laboratory, ECG and echocardiography) within 3–5 days after treatment start	38 (84)

IVIG, intravenous immunoglobulin; ECG, electrocardiography; ICU: intensive care unit; CRP, C-reactive protein; IVIG, immunoglobulin. *Dosage according to local decision.

Anti-inflammatory treatment comprised intravenous immunoglobulin 1–2 gram per kilogram bodyweight over 12–24 h given to all 42/45 (93%) but three children. Steroids were given in 38/45 (84%), of these 24/38% (63%) received methylprednisolone (10–30 mg per kg and day, over 3 days) and 16/38 (42%) prednisolone 2 mg/kg/day, two patients received both. Steroids were administered until fever cessation and then tapered over 2 weeks. Acetylsalicylic acid (aspirin) was given in all cases, 26/45 (58%) received high dosages (30 mg/kg/day) initially, followed by low dose (3–5 mg/kg/day) for 3 months in all but 6 patients, who received low dose aspirin for 6 weeks. For recurrent fever and persisting inflammation, 5/45 (11%) patients were treated with the recombinant Interleukin-1 receptor antagonist anakinra with 2–4 mg/kg/day over 3 days in 3 dosages and then reduced over 6 days, according to inflammation signs and symptoms. Baseline clinical and biochemical characteristics were indifferent in anakinra treated patients from the other PIMS patients. All but six children (87%) received prophylactic anticoagulation with enoxaparin subcutaneously. No thrombotic events were reported. Treatment response (decline of fever, symptoms and inflammation parameter) between 3 and 5 days after initiation of therapy was reported in 38/45 (84%). For three of the remaining seven patients the decision was made to administer another course of IVIG and four children received the above mentioned anakinra. All patients survived.

## Discussion

This study is a descriptive analysis of 45 consecutive patients with PIMS-TS admitted to nine pediatric departments in eastern Austria between March 2020 and May 2021. Almost all patients presented in reduced general condition with fever, rash and gastrointestinal symptoms. Importantly, three quarters of the patients had cardiac involvement including arterial hypotension, cardiogenic shock, and with signs and symptoms of myocarditis.

The majority of our patients were diagnosed with PIMS-TS following the local Austrian SARS-CoV-2 infection peaks between October 2020 and January 2021 and February to May 2021, before approval of the vaccination for children. Their prior COVID-19 infection was most likely caused by the SARS-CoV-2 wild virus type B1 from spring 2020, which was followed by the British variant (B1.1.7, Alpha) in autumn 2020. The Beta variant (B.1.351) was seen between January and March 2021, thereafter the Delta variant (B.1.617.2) took over in May 2021 (dominant until December 2021).

Patients with PIMS-TS in our cohort were older, had myocarditis and circulatory dysfunction, contrary to KD (8). Additionally, laboratory signs indicated general inflammation and cytokine release as reflected by increased CRP, IL-6 and ferritin, consistent with the PIMS-TS and MIS-C criteria, but again different to KD (3, 5, 13). The prominent gastrointestinal symptoms, which in our population often delayed diagnosis, may have been caused by a gastrointestinal epithelitis as described by Yonker et al. (14). During the study period high fever, reduced condition and abdominal pain in combination with a COVID-19 history increased the suspicion index of PIMS-TS diagnosis to initiate early effective treatment.

In our analysis 72% of the patients presented with arterial hypotension, abnormal cardiac troponin and almost all patients had significantly increased NT-proBNP, indicating relevant myocardial stress. The initial shock situation presumably was a combination of cardiac dysfunction due to myocarditis and vasoplegia because of inflammation. In a majority of patients echocardiography revealed left ventricular dysfunction and mitral valve regurgitation, comparable to the multicenter European cohort study by Valverde et al. who summarized 286 patients after PIMS-TS with shock, myocarditis and cardiac dysfunction. Their patients (93%) also had raised cardiac troponin suggesting myocardial injury in the course of PIMS-TS (8).

Most of our patients had a combination of increased cardiac troponins, NT-proBNP and inflammation parameters, which have been described to be highly suggestive for myocarditis (8). Also in children troponin I and T poses a valid parameter of myocardial injury (15, 16).

Myocarditis is an inflammatory disease of the heart muscle with diverse causes, such as immunological or viral, that cannot be differentiated easily (17). Of our patients, 82% had a positive COVID-19 history and SARS-CoV-2 antibodies, suggestive of an possibly ongoing immunological process. Although an investigation demonstrated widespread antigenemia in several organs in the absence of nasopharyngeal virus material weeks after SARS-CoV-2 infection (14), another study suggested that persistent antigenemia is not a common contributor to MIS-C (18). We speculate that our unvaccinated patients may have developed an immunologically triggered multi-organ inflammation with cardiac involvement and signs of myocarditis after infection with the mentioned SARS-CoV-2 variants.This would be consistent with international epidemiological studies from the United Kingdom, United States and France reporting PIMS-TS with myocarditis 4–6 weeks after local infection peaks. Their patients also had increased cardiac troponins and positive SARS-CoV-2 antibodies, but negative COVID-19 PCR, also indicating a post-viral immunologically triggered process followed by cardiac dysfunction (2, 19, 20). In contrast, Belhadjer et al. published a case series of 35 children with PIMS-TS and significant ventricular dysfunction (28% required extracorporeal membrane oxygenation) with only mild troponin elevation. However, 88% had positive COVID-19 PCR, indicating a rather direct viral induced myocarditis (7). It remains to be seen if later variants of the SARS-CoV-2 virus will also cause PIMS-TS or similar syndromes with cardiac affection.

The majority of our patients received anti-inflammatory treatment with corticosteroids, either prednisolone or methylprednisolone pulse therapy (dosages varied between the pediatric departments) and immunoglobulins. According to available data at the time of the study, management and treatment of patients with PIMS-TS was based on guidelines for related syndromes like KD, toxic shock syndrome and macrophage-activation syndrome (21). More recent literature confirmed a benefit of IVIG and steroids compared to IVIG alone with regard to cardiac function and general outcome (22, 23). One explanation for the good response to steroids might be biological similiarities to toxic shock syndrome, involving the T-cells, as recently described by Sacco et al. (24). Furthermore, recently appeared evidence supports early start of anti-inflammatory treatment (25, 26). Our patients showed excellent response with improved blood pressure to initial volume administration, immunomodulatory and anti-inflammatory treatment. This might be the reason why despite of a high proportion of patients with arterial hypotension (72%) only 40% required inotropes. All patients were discharged with improved cardiac function and general condition, no patient died. We confirm a high morbidity but low mortality in patients with PIMS-TS, as described in other cohorts (9, 27).

The long-term management after PIMS-TS in general and particularly in patients with coronary involvement is not well established. Physical sparing, sports ban for at least 3 months and regular follow-up by a pediatric cardiologist have usually been recommended. Follow-up investigations should comprise echocardiography and, in some cases cardiac MRI and cardiopulmonary exercise test before restarting physical activity (28).

Our study has some limitations. The data collection was retrospective but we went through much efforts to identify all patients presenting with PIMS-TS to the participating hospitals. SARS-Cov2 mutations were not documented consistently, and of the 9 patients with positive PCR incomplete information concerning Ct values are available. As this was an observational, retrospective study, diagnostic work up was per local institutional standards with excellent documentation. For uniform local data collection some of the results (such as ferritin) were only documented as “normal” or “abnormal,” which affected quality of analysis. Treatments were not standardized and therefore varied between the centers. Echocardiography and cardiac evaluation in the different departments was performed by pediatric cardiologists.

## Conclusion

In our analysis of unvaccinated Austrian children with PIMS-TS three quarters of the patients had cardiac involvement with significant myocarditis and circulatory dysfunction requiring inotropic support and intensive care management. Our data confirm the excellent response to intravenous immunoglobulins and steroids resulting in clinical improvement and discharge of the children with almost resolved cardiac signs. Follow-up of our patient series is ongoing to detect long term complications of this new disease entity. Further evaluation of pediatric patients with COVID-19 associated diseases is required to evaluate the impact of emerging virus variants.

**What is already known on this topic**

– Multisystem inflammatory syndromes in children can involve several organs. Kawasaki disease (KD) is a well-known inflammatory disease, where an acute vasculitis can cause a mild and transient myocarditis.
– A new COVID-19 associated multisystem inflammatory syndrome (PIMS-TS in Europe, MIS-C in USA) shows several similarities but also different features compared to KD, including the older age and more severe cardiac involvement.
– Several international investigations demonstrated a temporal association between local SARS-CoV-2 infection peaks with an increasing incidence of PIMS-TS cases.

**What this study adds**

– For a better understanding of this new disease it is useful to collect as much information as possible with regard to the considerable number of children who developed PIMS-TS associated cardiac impairment.
– Our study adds more valuable information concerning the clinical picture of PIMS-TS and signs of an associated myocarditis.
– This study contributes data from Middle Europe concerning PIMS-TS after infection with SARS-Cov-2 wild-virus, before availability of the vaccination for children.

**How this study might affect research, practice or policy**

The increasing evidence and understanding of the new PIMS-TS and its distinction from KD improves quality and tempo of diagnosis.

Because of the high prevalence of cardiac involvement in the course of PIMS-TS, early identification of children who require immediate cardiac (inotropic) support and anti-inflammatory treatment is crucial to improve outcome of this severe complication.

## Data Availability Statement

De-identified patient datasets will be available upon request to the corresponding author.

## Author Contributions

KT, BK, and IM-B conceived and designed the study. BK and KT conducted analysis and produced the tables. KT produced the initial draft, editing, and submission. CM did additional editing. All authors take full responsibility for data collection, data analysis, interpretation and submission of the study.

## Conflict of Interest

The authors declare that the research was conducted in the absence of any commercial or financial relationships that could be construed as a potential conflict of interest.

## Publisher’s Note

All claims expressed in this article are solely those of the authors and do not necessarily represent those of their affiliated organizations, or those of the publisher, the editors and the reviewers. Any product that may be evaluated in this article, or claim that may be made by its manufacturer, is not guaranteed or endorsed by the publisher.

## Abbreviations

PIMS-TS: Pediatric inflammatory multisysteme syndrome-temporally associated with SARS-CoV-2
MIS-C: multisystem inflammatory syndrome in children
KD: Kawasaki disease
VSD: ventricular septum defect
ECG: electrocardiography
MRI: magnetic resonance imaging
PCR: polymerase chain reaction
CRP: C-reactive protein
IL-6: Interleukin-6
NT-proBNP: N-terminal pro B-type natriuretic PEPTIDE
IQR: Interquartile ranges.
